# Light Availability and Patterns of Allocation to Reproductive and Vegetative Biomass in the Sexes of the Dioecious Macrophyte *Vallisneria spinulosa*

**DOI:** 10.3389/fpls.2019.00572

**Published:** 2019-05-03

**Authors:** Lei Li, Mingming Ding, Zhichun Lan, Yao Zhao, Jiakuan Chen

**Affiliations:** ^1^Jiangxi Province Key Laboratory of Watershed Ecosystem Change and Biodiversity, Center for Watershed Ecology, Institute of Life Science and School of Life Sciences, Nanchang University, Nanchang, China; ^2^National Ecosystem Research Station of Jiangxi Poyang Lake Wetland, Nanchang, China; ^3^Ministry of Education Key Laboratory for Biodiversity Science and Ecological Engineering, Institute of Biodiversity Science, Fudan University, Shanghai, China

**Keywords:** carbon limitation, dioecious plant, light intensity, plasticity, resource allocation, sexual dimorphism, submersed macrophyte

## Abstract

Environmental changes, e.g., eutrophication, in aquatic ecosystems can greatly alter light available to submerged macrophytes. In dioecious plants, given potential for sex-specific differences in resource requirements (i.e., high-carbon for seeds vs. high-nitrogen for pollen), females and males are expected to divergently adjust allocations toward resource acquisition structures when resources are limited during growth. Here, *Vallisneria spinulosa* was used as a representative dioecious submerged macrophyte to detect sex-specific responses to light limitation and assess whether sexual dimorphism varied with resource availability. Plants were grown under varying levels of light availability in nine outdoor mesocosms for 14 weeks. Late in the reproductive season, allocations to vegetative and reproductive traits for both sexes were determined and relative allocation to reproduction vs. vegetative growth was analyzed. Female and male reproductive plants differed in adjustments of resource allocation in response to light availability. Under low light, females showed a smaller reduction in allocation of resources to vegetative tissues and greater leaf area than males, suggesting female plasticity to increase carbon capture. Under low light, males showed a smaller reduction in reproductive allocation than females (flowers and inflorescences in males vs. fruits in females), suggesting that carbon limitation has greater impacts on sexual reproduction by females than males. Our study provides evidence of differences in reproductive costs and currencies for female vs. male reproduction in aquatic macrophytes, as *V. spinulosa* responded plastically to reduced light, with sexually dimorphic allocation strategies. Sex-related resource currencies are potentially important drivers for sex-specific variations in allocation patterns, with females safeguarding their vegetative carbon-rich biomass to satisfy future fruit and seed production.

## Introduction

Most flowering plants are hermaphroditic, with dioecy in only 5 to 6% of angiosperm species (Renner, 2014). Separate sexes have evolved repeatedly from hermaphroditic ancestors, in different lineages, probably in response to selection either for inbreeding avoidance (Charlesworth and Charlesworth, 1978) or sexual specialization (Charnov et al., 1976; Charlesworth, 1999). Transitions from hermaphroditism to dioecy are often related to evolution of sexual dimorphism. Once plants have evolved separated sexes, males and females have distinct roles; therefore, selection may favor divergence in life-history traits to optimize seed and pollen production (Barrett and Hough, 2013), presumably enabling sex-specific strategies of resource acquisition and reallocation.

Sexual dimorphism occurs in many dioecious terrestrial plants, with sexual morphs differing in: number, size and morphology of flowers and inflorescences; rates of growth, time to reproduction and timing of senescence; physiological traits, e.g., rates of photosynthesis and water uptake; and allocation toward life-history traits and anti-herbivore defenses (reviewed by Ågren et al., 1999; Dawson and Geber, 1999; Delph, 1999; Eckhart, 1999). Degree of sexual dimorphism varies among plant and animal species (Harris and Pannell, 2010; Laiolo et al., 2013; Tonnabel et al., 2014) and among populations within a species (Teder and Tammaru, 2005; Tonnabel et al., 2017).

Whereas sexual selection resulting from variation among individuals in mating success is the most likely cause of sexual dimorphism in animals (Ågren et al., 1999), evolution of sexually dimorphic characters in plants probably occurs due to differential resource requirements for reproduction. Female function (i.e., seed and fruit production) generally requires much carbon (Antos and Allen, 1990; McDowell et al., 2000), whereas male function probably has larger nitrogen requirements for pollen production (Harris and Pannell, 2008; Van Drunen and Dorken, 2012). Given female and male reproduction are differently limited by contrasting resources or “currencies,” selection on resource-harvesting traits likely differs between sexes, potentially eliciting sex-specific differences in “somatic cost of reproduction” for such different types of currencies (Obeso, 2002).

The differential plasticity hypothesis proposed that males and females differ in plastic responses to environmental factors, causing variation in degree of sexual dimorphism across environmental gradients (Delph and Bell, 2008). Divergent resource currency requirements for female vs. male reproduction may result in evolution of sex-specific plasticity in resource acquisition traits in response to environmental changes in resource availability (i.e., carbon and nitrogen). Previous studies examining the differential plasticity hypothesis focused on effects of nitrogen limitation from the perspective of relative resource investment in functionally different organs. For example, under low-nitrogen conditions, males likely benefit more than females from increasing allocation to belowground structures, as they harvest nitrogen needed for pollen production (Harris and Pannell, 2008; Teitel et al., 2016). However, some studies have also found that under dune soils, characterized by low-nitrogen contents, female plants showed greater belowground biomass than males (Sanchez-Vilas et al., 2012). If reproductive effort in females is limited more by carbon than nitrogen, females may exhibit plasticity in growth when exposed to carbon limitation to secure more carbon than males to ensure future carbon-rich reproduction. Females may also deploy sufficient resources to aboveground vegetative organs before involving carbon allocation toward flowering, as a way to maximize carbon acquisition. However, sex-specific variations in reproductive effort in response to a carbon limitation, which potentially limits female function, is not well understood (Tonnabel et al., 2017).

Submerged macrophytes play an important role in maintaining healthy aquatic ecosystems. Eutrophication and degraded underwater light climate is one of the fundamental reasons causing worldwide disappearance of submerged macrophytes (Schelske et al., 2010; Zhang et al., 2017). Variations in light reception influence the potential of a plant to capture carbon, which may limit plant growth and photosynthesis. Substantial phenotypic plasticity for resource capture is important for submerged macrophytes (Barrett et al., 1993; Going et al., 2008; Hyldgaard and Brix, 2012), enabling them to be more competitive (Chambers and Kalff, 1987). For instance, submerged species are highly responsive to light shortage and can have morphological and structural adaptations over the period of growth to optimize light utilization by increasing shoot length, decreasing branches, producing longer, wider and thinner leaves and changing resource allocation to various plant structures (Riis et al., 2012; Schneider et al., 2015; Chen et al., 2016). Numerous studies have been conducted to address the responses of submerged macrophytes to decreased light, albeit mostly from the perspective of growth conditions. To our knowledge, whether male and female plants are optimized by different resource allocation strategies in response to altered light availability is not well known. Thus, we herein address the responses of submerged macrophytes to limited light conditions from the perspective of sexual dimorphism.

In shallow freshwaters, substantial changes in light availability in populations of submerged macrophytes could be induced by eutrophication (Cao et al., 2011; Zhang et al., 2016). Plants generally respond plastically to changed resource availability during growth, even after reproduction commences. However, whether such a response is sexually dimorphic and reflects expectations based on potential existence of sex-specific resource currency requirements, remains to be investigated. Here, we used *Vallisneria spinulosa* as a representative dioecious submerged macrophyte to investigate potential responses in vegetative and reproductive traits to light availability and to evaluate patterns of sexual dimorphism, based on the differential plasticity hypothesis. In an aquatic mesocosm experiment that varied light availability of plants during their growing season, we tested three hypotheses: (1) females respond to reduced light by decreasing allocation toward aboveground growth less than males to satisfy carbon demands for future reproduction; (2) with increasing light limitation, females decrease allocation toward sexual reproduction more than males to ensure future capacity for carbon acquisition; and (3) sexual dimorphism in resource allocation is enhanced under stressful environmental conditions (e.g., limited light). Our results demonstrated that variations in light availability differentially affected males and females in their ability to perform photosynthesis, with implications for further understanding the effects of eutrophication on submerged macrophytes and also evolution of aquatic plants to varied environments.

## Materials and Methods

### Study Species

*Vallisneria spinulosa* S. Z. Yan (Hydrocharitaceae) is a dioecious, rosette-forming clonal submerged macrophyte with a hydrophilous pollination system. Light interception and utilization in *V. spinulosa* are determined by its long, strap-shaped leaves rather than by its short basal meristem stem; therefore, leaf growth impacts survival and reproduction. This plant commonly occurs in ponds, streams and lakes in mid-lower reaches of the Yangtze River in China (Wang et al., 2010) and grows well in waters with medium turbidity (Chambers and Kalff, 1987). It provides food for waterfowl, nursery habitats for fish and a substrate for invertebrates, and may modulate water quality (Wang et al., 2010).

In common with other *Vallisneria* species (Sculthorpe, 1967; Cox, 1988), female flowers develop underwater and are brought to the surface singly on elongated slender peduncles and at anthesis float on the surface. Male inflorescences (spathes) are located among bases of leaves with hundreds of packed male flowers. At maturity, male flowers are released to the water surface and aggregate around female flowers. Pollinated flowers are retracted underwater via a helix of peduncles of female flowers, where fleshy fruit develop. Clonal growth results from production of rosette-like ramet along elongating stolons and in the autumn, ramets produce several tubers that overwinter in sediment and germinate the following spring.

### Tuber Collection

Numerous *V. spinulosa* tubers were randomly collected (February 2017) from a large dioecious population at Bang Lake (115°55′–116°06′E, 29°11′–29°18′N; average water depth, 3.0 m) in Poyang Lake National Nature Reserve (Jiangxi Province, China, 115°55′–116°03′E, 29°05′–29°15′N). The tubers could not be sexed when they were collected. Tubers were cleaned, transported to the laboratory and kept in a water-filled plastic container maintained in darkness at 4°C for approximately 4 months.

### Experimental Design

To investigate sex-specific patterns of phenotypic plasticity in response to light availability and how sexual dimorphism in resource allocation varied across light gradients, an outdoor experiment was conducted in nine concrete mesocosms (2 × 2m, with water 1.6 m deep) at the Poyang Lake Laboratory for Wetland Ecosystem Research, Chinese Academy of Sciences (116°03′E, 29°26′N) located in Lushan City, China. Initially, 234 mature *V. spinulosa* tubers of uniform size (mean fresh mass ± SE = 0.906 ± 0.146 g; range of tuber mass = 0.722–1.335 g) were individually planted into plastic pots (23 cm diameter and 17 cm deep) containing a 4:1 mixture of Poyang Lake sediment and fine sand. Sediment was dried, ground and filtered through a sieve with a 0.5-cm mesh. Water (TN: 1.50 mg l^−1^; TP: 0.05 mg l^−1^; Chla: 6.6 μg l^−1^) was pumped from Poyang Lake and filtered (64-μm diameter net). There were three levels of light availability in a randomized block design, with each of the three blocks containing one replicate of each treatment. Six mesocosms were covered with black neutral shade nets to achieve the following light levels as percent of incoming light: 20% and 60% ambient light, representing low and medium light intensity, respectively. The other three mesocosms were unshaded and thus received 100% of ambient light, representing high light intensity. There were 234 pots randomly assigned to nine concrete mesocosms. For each light treatment, three replicate mesocosms were used and each mesocosm contained 26 pots (*n* = 78 pots/treatment). Each pot was tied with a nylon rope to a galvanized metal tube, suspended at 60 cm water depth. To renew water and maintain a consistent level, two or three times weekly, water from a common header tank was added to each mesocosm. Epiphyton on the leaves of *V. spinulosa* was removed with a soft brush every month to exclude shading of epiphyton on macrophyte. Plants grew in mesocosms for 14 weeks during summer and autumn (4 June to 10 September 2017). Sexual dimorphism is inconspicuous in pre-reproductive juveniles and plants can only be accurately sexed in *V. spinulosa* a few days after onset of flowering, based on spathe morphology. Therefore, because we used randomly chosen plants once tubers geminated, sex ratios of flowering plants in each treatment showed a strong male bias (average sex ratio = 2.87).

Photosynthetically active radiation (PAR) was measured with a Li-COR UWQ-192SA sensor coupled with a Li-1400 data logger (Li-Cor, Lincoln, NE, United States) at 10:00–12:00 under clear skies on 16 July 2017. PAR at water surface in low to high light regimes was 436.82 ± 6.45, 889.17 ± 16.45 and 1787.89 ± 18.75 μmol m^−2^ s^−1^ (mean ± SE, *n* = 9), respectively. For sediment in pots, contents of total N, total P and organic matter were 2.45 mg g^−1^, 0.73 mg g^−1^ and 6.61% (*n* = 3 pots).

### Allocation Measurements

At fruit maturation (98 days after planting), above- and below-ground portions of well-developed, intact plants of both sexes in each mesocosm were harvested. Final numbers of harvested male plants were 59, 44, and 35 for low, medium, and high light treatments, respectively, and those of harvested female plants were 19, 22, and 10. Because some stunted plants grew very poorly and did not flower and others were grazed by aquatic insects, only plants that grew vigorously and flowered were harvested. After removal from sediments, plants were carefully washed with tap water.

To assess male and female resource allocations, traits for both growth and reproduction were measured. Plant height, ramet number, shoot biomass (i.e., leaf biomass), leaf number and leaf area are key traits related to plant size and competitive interactions for light. Plant height was measured as length of the longest leaf. Total number of leaves for 10–15 plants per treatment were recorded and entire leaf surface area for each plant determined with an AM-350 Leaf Area Meter (ADC Bioscientific, Hoddesdon, United Kingdom). To determine percentage of flowering ramets, total and flowering ramet numbers for each plant were counted. Number of tubers and for males, number of spathes (including intact spathes and pedicles of spathes that had fallen off) and for females, number of fruits, were counted. Every plant was separated into three components: vegetative biomass (including leaves, roots, and stolons); sexual reproductive biomass (spathes or peduncles and fruits); and clonal reproductive biomass (tubers). To determine dry mass for each component, samples were dried at 75°C for 3 days and then weighed on a SI-114 four-decimal place gram balance (Denver Instrument, Denver, CO, United States). Because dispersal of pollen from some male spathes occurred when plants were harvested, we randomly sampled 10 non-dehiscent, mature male spathes from five male plants within each light regime and weighed each spathe individually after drying. Data from these 10 male spathes obtained in each light regime were averaged and multiplied by the total number of spathes produced per male to estimate biomass of sexual reproductive tissues of male plants for each light regime. In this study, dry mass of leaves, roots, and stolons (i.e., non-reproductive parts) was combined as vegetative investment (i.e., vegetative growth). Sexual reproductive effort was calculated by dividing total mass of male spathes or female peduncles and fruits by resources allocated to the entire plant. Allocation to clonal reproduction was determined by dividing tuber biomass by total biomass.

### Statistical Analyses

To investigate if sexual dimorphism in resource allocation varied with light availability, a Restricted Maximum Likelihood (REML) linear mixed model was used. For this model, sex and light treatment were fixed factors and block was random factor, with each life-history trait analyzed independently. For all traits, the interaction between sex and light pointed to significant effect of light on sexual dimorphism. *Post hoc* comparisons among groups used a Tukey honestly significant difference (HSD) test procedure (α < 0.05). To investigate our differential plasticity hypothesis, for each sex, relative allocation to vegetative and sexual reproductive biomass was calculated as percentage change in biomass under low light compared to mean allocation under high light. Tests of equality of means between sexes were done with a Student’s *t*-test (α = 0.05). All analyses used SPSS software, version 19.0 (SPSS Inc., Chicago, IL, United States).

## Results

### Sexual Dimorphism in Aboveground Growth and Reproductive Traits

**FIGURE 1:**
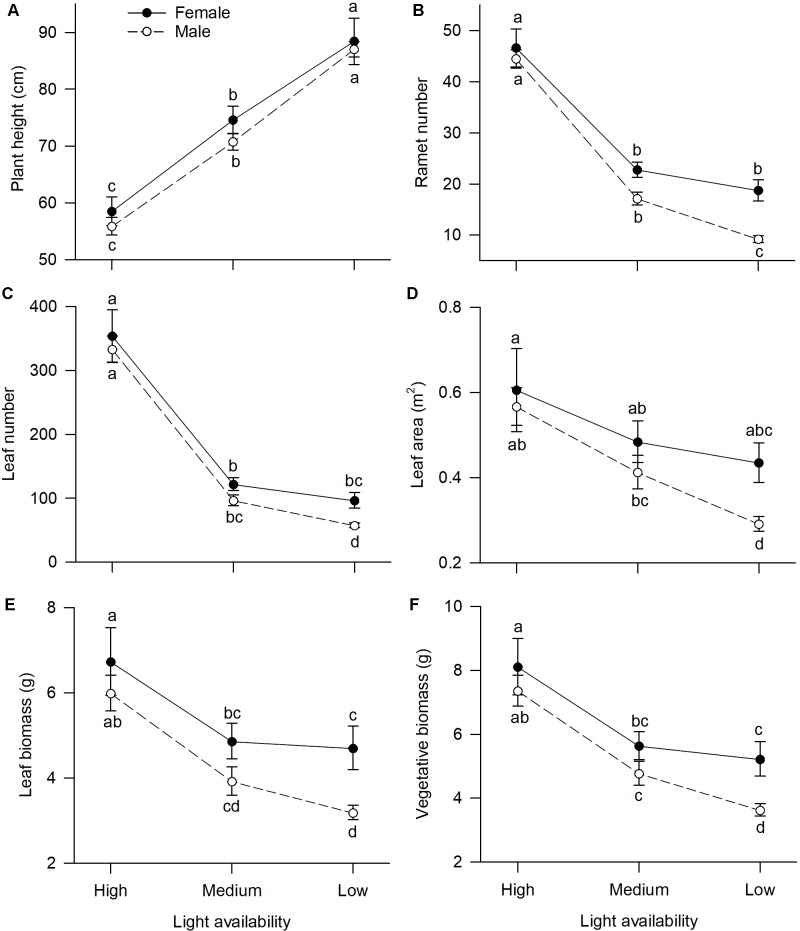
Effects of light availability on allocation to vegetative tissues for both males and females of *V. spinulosa* at reproductive maturity: **(A)** plant height, **(B)** ramet number, **(C)** leaf number, **(D)** leaf area, **(E)** leaf biomass, and **(F)** vegetative biomass. Letters denote significant difference (*P* < 0.05), using Tukey HSD comparisons. Error bars show ± SE. Curves are usually used to display differences between sexes under different environmental conditions (Harris and Pannell, 2008; Hesse and Pannell, 2011).

Of traits representing allocation to growth, all but one, “plant height,” had significant sexual dimorphism (Table 1). Under high or medium light, all vegetative traits showed a non-significant tendency to be greater in females compared to males (Figure 1). Under low light, females exhibited significantly greater investment in leaf and vegetative biomass, ramet number, leaf number, and leaf area than males (Figure 1B–F).

**Table 1:** Results of the Restricted Maximum Likelihood (REML) linear mixed model analyses examining effects of sex and light treatment on traits of *V. spinulosa*.

Traits	Results	Source of Variation		
		Sex (*df* = 1)	Light (*df* = 2)	Sex × Light (*df* = 2)
**Vegetative traits**				
Plant height	*F*	2.575	96.924	0.207
	*P*	0.110	**<0.001**	0.813
Ramet number	*F*	19.331	174.326	2.337
	*P*	**<0.001**	**<0.001**	0.100
Leaf number	*F*	6.691	176.379	0.237
	*P*	**0.010**	**<0.001**	0.789
Leaf area	*F*	4.262	15.660	0.546
	*P*	**0.041**	**<0.001**	**0.044**
Leaf biomass	*F*	18.980	28.092	0.792
	*P*	**<0.001**	**<0.001**	**0.019**
Vegetative biomass	*F*	15.194	40.126	0.879
	*P*	**<0.001**	**<0.001**	**0.023**
**Reproductive traits**				
Flowering ramet number	*F*	29.442	48.428	4.465
	*P*	**<0.001**	**<0.001**	**0.013**
Percentage of flowering ramet	*F*	104.251	5.924	7.965
	*P*	**<0.001**	**0.003**	**<0.001**
Spathe/fruit number	*F*	55.715	20.717	7.863
	*P*	**<0.001**	**<0.001**	**0.001**
Tuber number	*F*	0.791	62.656	0.044
	*P*	0.375	**<0.001**	0.957
Sexual reproductive effort	*F*	68.400	10.920	5.682
	*P*	**<0.001**	**<0.001**	**0.004**
Clonal reproductive effort	*F*	1.124	1.781	4.391
	*P*	0.290	0.171	**0.014**

*Bold values are significant at P < 0.05.*

**FIGURE 2:**
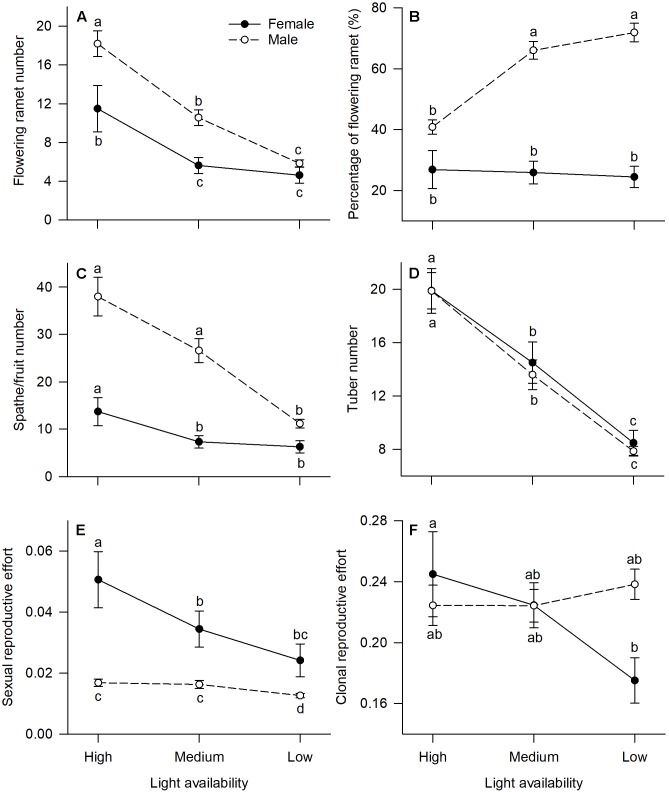
Effects of light availability on allocation to reproductive tissues for males and females of *V. spinulosa* at reproductive maturity: **(A)** flowering ramet number, **(B)** percentage of flowering ramet, **(C)** spathe/fruit number, **(D)** tuber number, **(E)** sexual reproductive effort, and **(F)** clonal reproductive effort. For each individual sexual and clonal reproductive efforts were calculated as the ratio between dry mass of the respective tissues (male: spathes; female: fruits) and total biomass. Letters denote significant difference (*P* < 0.05), using Tukey HSD comparisons. Error bars show ± SE. Curves are usually used to display differences between sexes under different environmental conditions (Harris and Pannell, 2008; Hesse and Pannell, 2011).

All measured reproductive traits, except those reflecting allocation toward clonal propagation (tuber number, clonal reproductive effort) displayed significant sexual dimorphism (Table 1). Males had a higher percentage of flowering ramets than females, but only in medium or low light treatments (Figure 2B). Males produced more flowering ramets and inflorescences than females under high or medium light (Figure 2A,C), but there was no significant difference between sexes under low light. In contrast, females invested proportionally more resources in sexual reproductive structures than males across all light treatments (Figure 2E).

### Sex-Specific Plasticity in Response to Variable Light

Light availability differentially affected males and females both in leaf biomass (sex × light interaction: *P* = 0.019; Table 1 and Figure 1E) and vegetative biomass (sex × light interaction: *P* = 0.023; Table 1 and Figure 1F). Males and females reduced leaf and vegetative biomass with decreasing light intensity; however, males did so more than females (Figure 1E,F). Leaf area also exhibited sex-differential plasticity in response to light availability (sex × light interaction: *P* = 0.044; Table 1 and Figure 1D). Whereas female leaf area was independent of light availability, males decreased their leaf area under low light (Figure 1D). Males and females both increased plant height with decreasing light (Figure 1A), whereas ramet number and leaf number had an opposite pattern (Figure 1B,C). Light availability did not affect sexual dimorphism in plant height (sex × light interaction: *P* = 0.813; Table 1 and Figure 1A), ramet number (sex × light interaction: *P* = 0.100; Table 1 and Figure 1B), and leaf number (sex × light interaction: *P* = 0.789; Table 1 and Figure 1C).

In both males and females, flowering ramet and inflorescence number were significantly reduced in low light (Figure 2A,C), but males had steeper decreases in flowering ramets (sex × light interaction: *P* = 0.013; Table 1 and Figure 2A) and inflorescences (sex × light interaction: *P* = 0.001; Table 1 and Figure 2C) than females, as light intensity decreased. Number of tubers in both sexes was reduced similarly with decreasing light (sex × light interaction: *P* = 0.957; Table 1 and Figure 2D).

### Growth vs. Reproductive Allocation

**FIGURE 3:**
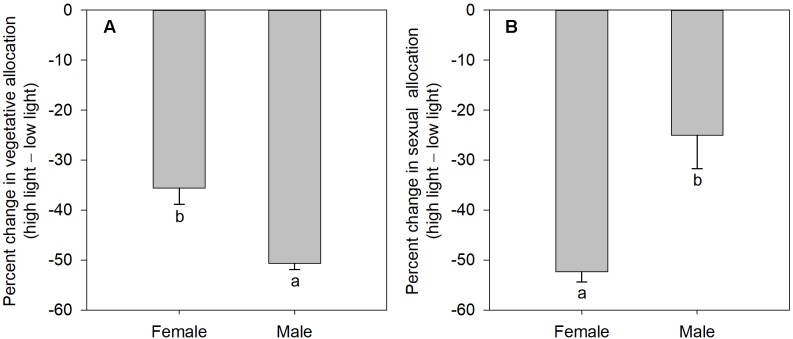
The mean percentage change in vegetative and sexual reproductive tissues under low light regime relative to their average allocation under high light regime; values are means ± SE, for males and females of *V. spinulosa*. **(A)** Vegetative and **(B)** sexual reproductive allocations were calculated as percentage change in biomass of the respective tissues compared to the mean allocation to these tissues under high light regime for each sex. Values with different letters denote statistically significant differences between sexes (*P* < 0.05), calculating using likelihood ratio test.

The degree of sexual dimorphism in sexual reproductive effort (proportion of biomass allocated to sexual reproductive structures) varied with light treatment (sex × light interaction: *P* = 0.004; Table 1 and Figure 2E). Both males and females displayed plastic responses in their relative allocation to sexual reproductive vs. vegetative tissues; this ratio was significantly lower in low light for both sexes (Male: *post hoc* comparison: *P* = 0.007; Female: *post hoc* comparison: *P* = 0.047; Figure 2E). However, decreased light caused females to reduce relative allocation to sexual reproduction more than males (Figure 2E). Vegetative biomass tended to decrease less in females than in males under lower light levels (Figure 3A), whereas allocation toward sexual reproductive biomass under lower light levels tended to decrease more in females than in males (Figure 3B). Whereas females reduced clonal reproductive effort under limited light (*post hoc* comparison: *P* = 0.028; Figure 2F), males maintained the same ratio among all light conditions (*post hoc* comparison: *P* = 0.564; Figure 2F).

## Discussion

Effects of altered light availability on performance of submerged macrophytes are well known (e.g., Xie et al., 2007; Riis et al., 2012; Yuan et al., 2012; Eller et al., 2015), but sex-specific plasticity in sexual dimorphism in response to varied light levels over an entire growing season has not been well characterized. The present study provides evidence supporting the differential plasticity hypothesis in aquatic environment, particularly in the context of sex-specific resource requirements, with insights into evolution of sexual dimorphism in dioecious macrophytes. Reproductive males and females differentially adjusted resource allocation to reproduction and aboveground vegetative growth in response to decreased underwater light availability. Whereas females showed a greater allocation of resources to vegetative tissues and leaf area than males under limited light, males showed a smaller reduction of resource allocation to sexual reproduction than females. Because carbon acquisition is more critical for female reproduction, under conditions of light limitation females are expected to decrease their reproductive allocation more than males, as a way to conserve resources for vegetative organs to ensure their carbon needs for future reproduction. Relevant results can help further understanding of the effects of eutrophication on submerged macrophytes and evolution of aquatic plants to varied environments.

### Plastic Resource Allocation With Varied Light

In our experiment, resource allocation in *V. spinulosa* was highly responsive to a change in light availability. Although various macrophyte species may respond differently to light deficiency (Chambers, 1987), decreased light in water often reduces coverage and decreases growth of macrophytes in the littoral zone and vice versa (*Vallisneria americana*, French and Moore, 2003; *Vallisneria natans*, Chen et al., 2016). In our experiment, both vegetative and reproductive traits tended to decrease as light decreased from 100% to 20% incident light. Unlike canopy formers (e.g., *Potamogeton maackianus*), *V. spinulosa*, being rosette-forming, cannot rapidly elongate to reach the water surface where light availability is high. Thus, aboveground vegetative growth (leaf biomass) of *V. spinulosa* declined in limited light condition, as also reported in *V. americana* (French and Moore, 2003) and *V. natans* (Fox et al., 2013) in adaptation experiments to shading.

A plastic response to decreased light in *V. spinulosa* involved linear increases in plant height through leaf elongation, decreased ramet number and leaf number and thereby decreased leaf area. Thus, reproductively mature plants showed less lateral growth and more elongated growth with decreasing light gradients, resulting in a shift of leaf area into better illuminated areas (Valladares and Niinemets, 2008). Such a plastic response has the same effect as foraging concept in most herbaceous terrestrial plants with erect stems, which hypothesizes that plants develop longer stems and less branches in low-light conditions to improve resource acquisition and reduce respiratory cost (de Kroon and Hutchings, 1995). Greater resource allocation to vertical growth also involved a trade-off, causing decreased resource allocation to reproduction, as reported (Harper, 1977), but decreasing total plant biomass. In the present study, plants also responded plastically to reduced light by altering resource allocation to reproductive traits. A negative effect on sexual reproduction is likely to influence seed dispersal, re-establishment after disappearance and may reduce resistance to other environmental stressors, due to lower genetic diversity (Boedeltje et al., 2008).

### Evidence of Differential Plasticity Hypothesis in Macrophytes

Our study highlighted flexibility of plants in resource allocation and how reproductive male and female plants responded plastically to light stress with resource allocation that maximized fitness. The differential plasticity hypothesis has been tested in several terrestrial plants (e.g., *Silene latifolia*, Lovett Doust et al., 1987; *Mercurialis annua*, Harris and Pannell, 2008; *Rumex hastatulus*, Teitel et al., 2016), with mixed results. Regarding the differential plasticity hypothesis, we emphasized plant flexibility in responding to local environments, particularly in varying allocation to growth vs. reproduction. Greater sexual dimorphism in vegetative tissues and smaller sexual dimorphism in resource allocation to sexual reproduction for dioecious *V. spinulosa* under low light was consistent with the differential plasticity hypothesis.

Flexible reproductive strategies are likely an adaptive advantage in aquatic environments, which often have strong spatio-temporal heterogeneity (Eckert et al., 2016). In aquatic ecosystems, environmental heterogeneity, e.g., eutrophication, can strongly affect access to light for submerged macrophytes, similar to that we manipulated in our study. We inferred plasticity in sexual dimorphism is advantageous to aquatic plants in the face of adverse environmental conditions caused by eutrophication, such as high turbidity and low irradiance penetration. In accordance with the differential plasticity hypothesis, males and females of *V. spinulosa* differentially adjusted their relative allocation of resources among vegetative and reproductive tissues with decreasing underwater light availability, reflecting sex-specific adaptation. Divergent plastic responses of male and female plants in vegetative and reproductive traits implied a potential reason for intraspecific variation in sexual dimorphism in resource allocation under light limitation (i.e., more stressful environment).

More specifically, decreased light for a submerged plant during its life cycle, as in our study, could be induced by re-suspension of sediment, variations in periphyton and phytoplankton abundance, or by concentrations of dissolved materials due to nutrient enrichment. If resource limitation elicits sex-specific plastic responses, spatial variation in this resource will cause spatial variation in degree of sexual dimorphism (Barrett and Hough, 2013). There is also ample evidence that higher reproductive expenditure and/or greater sensitivity to stress in females should result in more male-biased sex ratios among populations of a species along gradients of resource availability (e.g., Pickering and Hill, 2002; Li et al., 2007). Our results reflect environmental variation between sexes in their allocation to vegetative tissues and reproduction and provide support for differential plasticity contributing to variations in sexual dimorphism in aquatic macrophytes. A growing evidence from studies of sexual dimorphism under varying resource levels supported the differential plasticity hypothesis (Harris and Pannell, 2008; Hesse and Pannell, 2011; Teitel et al., 2016; Li et al., 2019). However, details of how resource currencies are differently reallocated from sources to sinks and how patterns of variation in sexual dimorphism are driven by different resource currencies, remain largely unknown.

### Sex-Specific Strategies of Resource Allocation

Our results illustrated that high levels of plasticity in response to the environment affected traits that can be sexually dimorphic in dioecious plants; therefore, indices of dimorphism themselves become plastic. In *V. spinulosa*, how resource currencies were reallocated from sources to sinks was altered in response to light limitation, consistent with responses in dioecious *M. annua* (Tonnabel et al., 2017). Sex-specific allocation patterns in *M. annua* vary temporally and also respond to environmental heterogeneity (Harris and Pannell, 2008). Males and females used disparate resource allocation strategies, exhibiting sex-specific resource trade-offs, which can involve disparate resource currencies in females and males. In many dioecious plants, female reproductive costs may exceed those of males, due to high investments in carbon-rich fruits and seeds (Barrett and Hough, 2013). In particular, flowering in many submerged macrophytes requires considerable investment in reproductive accessory structures, e.g., production of peduncles in female *V. spinulosa*, to bear flowers at or above the water surface. Reproductive costs result in physiological trade-offs in resource distributions, which can influence future vegetative growth and reproduction. Therefore, females of *V. spinulosa* are expected to have stronger trade-offs with other life-history traits, due to their typically higher investment in sexual reproduction.

Our results conformed to expectations arising from the differential plasticity hypothesis; namely, that females maximize allocation to aboveground growth under conditions of light limitation more than males, as carbon acquisition is more critical for female reproduction. This pattern was due to a stronger reduction of allocation to reproductive tissues in females than in males and a smaller reduction of allocation to vegetative tissues in females than in males when light availability decreased. This points to existence of a greater trade-off in females than males between current and future reproduction in terms of carbon; with limited light, females decreased sexual reproductive effort more than males. This response may be due to a difference in resource currencies that limit female vs. male functions, e.g., carbon may limit reproduction by females more than that by males (Antos and Allen, 1990; McDowell et al., 2000). Perhaps carbon limitation causes a smaller decrease in allocation to carbon-harvesting organs (i.e., shoot and leaves) in females than in males, as females probably safeguard capacity to acquire carbon for future seed and fruit production.

Consistent with our results, similar evidence on sex-specific resource (carbon) budgets and differential allocation strategies in response to low light have been reported. For example, females reduced their allocation to vegetative growth in response to competition for light less than males (Lovett Doust et al., 1987; Tonnabel et al., 2017), perhaps due to a higher total carbon cost for female reproduction. In contrast, in some studies, females experienced a steeper or similar decrease in resource allocation to aboveground biomass in response to reduced access to light (Hesse and Pannell, 2011; Labouche and Pannell, 2016). These observations were contrary to the expectation, on the basis of a differential somatic cost of carbon between sexes, that females reduce aboveground biomass less than males when carbon becomes limiting. Therefore, our study on submerged macrophyte added to growing evidence providing support for a stronger somatic cost of carbon in females, potentially causing a sex-specific plastic response to limited carbon.

## Conclusion

In conclusion, our results highlighted that males and females have different strategies of resource allocation in response to carbon limitation. A decrease in light availability led to a smaller decrease in allocation to carbon harvesting organs in females than in males for the sake of maintaining a capacity for carbon acquisition for later seed and fruit production. Limited light condition caused a smaller reduction of reproductive allocation in males than in females, suggesting that the production of reproductive organs in females is more carbon-limited than in males (fruits in females vs. flowers and inflorescences in males). Eutrophication and degraded underwater light climate is a fundamental cause of worldwide declines in submerged macrophytes (Zhang et al., 2017). Therefore, investigating sex-specific strategies of resource allocation in response to varying light availability is essential for better understanding the adaptation and evolution of submerged macrophytes in freshwater habitats.

## Author Contributions

LL designed the study, executed the experiments and wrote the first draft of the manuscript. LL and MD conducted the statistical analyses. JC, ZL, and YZ provided the scientific advice.

## Conflict of Interest Statement

The authors declare that the research was conducted in the absence of any commercial or financial relationships that could be construed as a potential conflict of interest.
